# High-Intensity Interval Training for Overweight Adolescents: Program Acceptance of a Media Supported Intervention and Changes in Body Composition

**DOI:** 10.3390/ijerph13111099

**Published:** 2016-11-08

**Authors:** Sabine Herget, Sandra Reichardt, Andrea Grimm, David Petroff, Jakob Käpplinger, Michael Haase, Jana Markert, Susann Blüher

**Affiliations:** 1 Integrated Research and Treatment Center (IFB) for Adiposity Diseases, University of Leipzig, Leipzig 04109, Germany; reichardt.sandra@gmail.com (S.R.); andrea.grimm@medizin.uni-leipzig.de (A.G.); david.petroff@zks.uni-leipzig.de (D.P.); jakob.kaepplinger@medizin.uni-leipzig.de (J.K.); jana.markert@uni-leipzig.de (J.M.); 2 Faculty of Architecture and Social Sciences, University of Applied Sciences Leipzig (HTWK), Leipzig 04315, Germany; 3 Clinical Trial Centre, University of Leipzig, Leipzig 04109, Germany; 4 CityBootCamp Outdoor Fitness Training, Leipzig 04103, Germany; info@citybootcamp.de; 5 Department of Pediatrics, University of Halle-Wittenberg, Halle 06108, Germany

**Keywords:** overweight, obesity, adolescents, high intensity interval training (HIIT), new media intervention

## Abstract

High-intensity interval training (HIIT) consists of short intervals of exercise at high intensity intermitted by intervals of lower intensity and is associated with improvement of body composition and metabolic health in adults. Studies in overweight adolescents are scarce. We conducted a randomized controlled trial in overweight adolescents to compare acceptance and attendance of HIIT with or without weekly motivational encouragement through text messages and access to a study website. HIIT was offered for six months (including summer vacation) twice a week (60 min/session). Participation rates were continuously assessed and acceptance was measured. Clinical parameters were assessed at baseline and after six months. Twenty-eight adolescents participated in this study (age 15.5 ± 1.4; 54% female). The standard deviation score for body mass index over all participants was 2.33 at baseline and decreased by 0.026 (95% CI −0.048 to 0.10) units, *p* = 0.49. Waist to height ratio was 0.596 at baseline and decreased by 0.013 (95% CI 0.0025 to 0.024), *p* = 0.023. Participation within the first two months ranged from 65% to 75%, but fell to 15% within the last three months. Attendance in the intervention group was 14% (95% CI −8 to 37), *p* = 0.18, higher than the control group. Overall program content was rated as “good” by participants, although high drop-out rates were observed. Summer months constitute a serious problem regarding attendance. The use of media support has to be assessed further in appropriately powered trials.

## 1. Introduction

Physical inactivity is a worldwide public health problem. It is a crucial risk factor for the development of obesity and associated metabolic and mental diseases [1]. The WHO guidelines of 2010 advise children and adolescents to perform physical activity of moderate to vigorous intensity for at least 60 min per day [2]. However, the percentage of inactive adolescents is also very alarming, with 80% of teenagers aged 13–15 years failing to meet physical activity recommendations [3,4].

This puts adolescents at high risk for the development of obesity and associated sequalae and emphasizes the need to foster physical activity, which will improve cardio-respiratory function and daily energy expenditure. So far, a variety of exercise programs have been offered to and evaluated in adolescents [5,6,7]. Recent studies have reported on high-intensity interval training (HIIT) regarding the improvement of metabolic health and cardiovascular risk factors in adolescents [8,9]. Generally, HIIT is a training method that includes short time periods of high intensive workout with an achievement of an 80% to 95% maximal heart rate followed by active recovery phases at a 70% maximal heart rate. Enjoyment, implementation, fidelity, and attendance of HIIT have to be monitored, as this novel training method could represent an important alternative to endurance training [9,10,11,12].

Therefore, a program offering HIIT to overweight adolescents including media support (text-messages and emails through a study website) to improve program attendance was developed by our group. The HIIT method consists of intervals of physical exercise, which comprises both strength and endurance. These two components have been shown to have beneficial effects on cardiovascular parameters, mitochondrial function in skeletal muscle, endothelial function, and aerobic work capacity by increasing insulin sensitivity and improving the lipid profile in adults and young females [13,14,15]. The aim of this paper is to analyze acceptance and attendance of HIIT in overweight adolescents. Anthropometric, physiological, biochemical, and metabolic study outcomes are presented in a separate study [16].

## 2. Materials and Methods

### 2.1. Study Design

This study was conducted as a randomized controlled trial to test feasibility, attendance, and acceptance of the media-supported “moveHIT” program. A sample size of 70 participants (35/treatment groups) was chosen based on the expectation that the sample standard deviation would be 6 for the number of sessions attended (see endpoints below). This would imply that the confidence interval describing the attendance would have a width of four sessions (10 percentage points) and permit a sufficiently accurate estimate of attendance rates. Subjects were randomized to two intervention treatment groups (i.e., one intervention group without media support and one intervention group with regular media support to increase participation). Reporting of this trial is in accordance with the 2010 CONSORT statement [17]. MoveHIT was registered retrospectively in the German Clinical Trials Register in 2014 (DRKS00006781), available in the International Clinical Trials Registry Platform of the WHO. Ethics approval of this study was obtained from the Ethical committee of the Medical Faculty of the University of Leipzig, Germany (AZ 261-13-26082013). Participants and their legal guardians provided informed written consent prior to the baseline examination. The study was performed in accordance with the Declaration of Helsinki and is based on principles of Good Clinical Practice (GCP).

### 2.2. Participants

Overweight adolescents aged 13–18 years (BMI > 90th percentile according to German reference values [18]) were eligible for participation. Twenty-eight adolescents provided complete baseline data.

Pregnancy and serious orthopedic impairment were exclusion criteria. Randomization was performed using prepared lists stratified by sex. There was no influence on the randomization procedure by the staff. Thus, the difference between both groups reflects the impact of the media support on training adherence, free of bias induced by problem awareness.

### 2.3. Intervention

Both groups received the same kind of high intensity interval training (HIIT) for 60 min twice a week that could be chosen from three available sessions per week (on weekdays) at the same time each afternoon (with approximately 8 to 15 participants each session) and lasted six months. Both intervention and control group attended the same training sessions due to the lower number of participants recruited into the study. The *intervention* group received two text-messages weekly and an email every other week consisting of relevant program information and tailored feedback to the participant (Table 1).

Each HIIT training session consisted of a 10 min warming-up period which aimed to have participants reach 50%–60% of their maximal heart rate (HR_max_), which was followed by alternating bouts of high-intensity exercise at 80%–95% of HR_max_ with active breaks at 50%–60% of HR_max_ in between the intervals. HR_max_ of the adolescents was determined in a 6-min run performed with maximal individual effort. Adolescents had to run for 6 min while wearing heart monitors and were instructed to strive for the maximal distance possible for them. Maximal heart rate during the run was recorded and subsequently used as HR_max_ [19,20]. HR was determined by using heart rate monitors (Polar^®^, Büttelborn, Germany) throughout the session and peak HR_max_ was protocoled after the session.

Content of the warming-up period were active games. High-intensity exercise included Tabata interval training with 20 s of high-intensity activity, such as running, followed by 10 s of active recovery such, as low volume strength exercises [21]. Protocols of intensity by Gibala were also applied [22], where 8–10 cycles of 60 s of sprinting were followed by 75 s of rest or active recovery. Participants were asked to check their heart rate monitors during the session to ensure training was conducted at the right intensity level. A cooling-down period of 5 to 10 min terminated the session. The training was implemented as an outpatient physical activity program over a period of six months, carried out in open spaces such as parks in the city of Leipzig, Germany by licensed sport scientists.

Media support of the intervention group consisted of regular text messages to encourage participants to attend the training session. They were sent out twice weekly and contained information such as “Dear (participant’s name), glad that you attended the last training session. You burned…calories! Great work. Hope to see you at the next session on…!” Another version would be: “Hello (participant’s name), we noticed that you did not attend the last training session. We hope you are going to attend tomorrow. Regular physical activity can help you reach your fitness goals!” Generally, the text messages were tailored and consisted of a greeting, motivation, and reminder of the next training session. Additionally, the intervention group also received emails twice a week containing information on training and physical activity. The intervention group had access to the emails by login into the study website. These emails were structured according to the social-cognitive theory by Bandura [23] and addressed the constructs of attitude formation, outcome expectations (regarding physical activity), strategies for self-regulation and overcoming relapse, seeking social support, and self-efficacy over the time course of six months (see Table 1). Text messages and emails were sent according to a standardized manual and staff had been trained to use standardized procedures when sending out text messages or email feedback.

### 2.4. Measurements and Study Endpoints

Study visits included one baseline visit at our study center after providing written agreement by participant and legal guardian and one closing visit after the end of the intervention.

The primary study endpoint was the attendance to the HIIT program measured by the number of sessions attended. The intervention group who received motivational text messages and had access to a password protected website for personal feedback was compared to the control group regarding program attendance.

Secondary endpoints included anthropometric and metabolic measurements, which were obtained by the same pediatrician at baseline and at follow up. For measurement of body height the digital stadiometer “Dr. Keller III” was applied, and body weight was determined by a digital scale (SECA^®^-scale, Vogel & Halke GmbH, Hamburg, Germany; precision ±100 g) [24]. After calculation of Body mass index (BMI), these data were standardized to age and sex of the children by applying German reference data [17], and were calculated as standard deviation of body mass index BMI-SDS according to the LMS method [25].

A flexible, non-elastic band was used to measure waist and hip circumferences: waist circumference was measured as the smallest abdominal girth between the lowest rib and the upper anterior iliac spine; hip circumference was defined at the maximal buttock circumference and was measured horizontally [26]. The skinfold thickness equation by Slaughter [27], where measurements of triceps, biceps, subscapular, and suprailiacal skinfolds were made in triplicate using a caliper (Holtain skinfold caliper, Crosswell, UK), was applied to determine the body fat percentage. Maturation status was defined according to Tanner stages.

### 2.5. Questionnaires

Daily physical exercise/sedentary behavior, health-related quality of life, self-efficacy, internalization of stigmatization, perceived social support, and outcome expectations over physical activity were measured via validated questionnaires for adolescents at baseline and after the intervention [28,29,30,31,32,33]. Participants were also asked to evaluate the overall program, exercise content, and trainers in a questionnaire with a Likert-scale from 1 (liked it very much) to 10 (did not like it at all) at the follow-up visit at the study center. Dietary behavior was not analyzed due to the primary focus of the study being on feasibility and program attendance.

### 2.6. Statistical Analyses

Descriptive analyses of data were performed using SPSS, version 20.0 (IBM Corp. Released 2011. IBM SPSS Statistics for Windows, Version 20.0. IBM Corp., Armonk, NY, USA). Means and standard deviation were calculated. The number of sessions attended by each participant was compared between the two groups using a *t*-test. The choice of a *t*-test was made at the planning stage although a skewed distribution was expected because the benefits of measuring the endpoint on a metric scale were considered large and the slight problems associated with the skewed distribution are known to be small for sufficient sample size [34,35]. We chose not to deviate from the planned primary analysis although the sample size could not be reached, but performed the non-parametric Wilcoxon-Mann-Whitney U test as a sensitivity analysis. A survival analysis was performed analyzing the event “drop-out from the program”. The point of time of drop-out was taken to be the last session attended. A Pearson correlation was computed to analyze program evaluation in relation to program attendance. Baseline and follow-up data were analyzed using R version 2.14 (Foundation of Statistical Computing, Vienna, Austria) [36]. A two sample paired *t*-test between the baseline and the follow up data was performed for anthropometric parameters where the means of missing data were treated as having remained unchanged but assuming their variance is comparable to that of the non-missing data. All values were considered to be significant at the *p* ≤ 0.05 level.

## 3. Results

### 3.1. Recruitment

Due to difficulties in recruitment, only 28 adolescents with a mean age of 15.5 ± 1.4 years participated in this study (54% females). They were randomized to control (*n* = 14) and intervention groups (*n* = 14). After program completion 20 participants were available for follow-up visits, 8 were lost to follow-up visits due to time constraints because of school work, apprenticeship obligations, and lack of interest. Nine (32%) of them were former participants of an outpatient obesity therapy program KLAKS, five (18%) were contacted through friends, three (11%) enrolled after finding a program flyer at a school club, and five responded to magazine reports and advertisements (18%). The remaining participants were enrolled by contact through teachers and social workers. Due to the lower number of participants recruited this feasibility study was underpowered. 

### 3.2. Leisure Time Habits and Physical Activity Enjoyment

At baseline, only 21% (6 out of 28) of participating adolescents met the recommendations of 60 min of daily physical activity over the course of a normal week. Furthermore, 43% of participants were aware of the associated benefits (reduced risk of illness, increased mental well-being, increased fitness, etc.) of regular physical activity. After the program, 30% of participants (6 out of 20; partially overlapping participants), who attended the follow-up examination, were physically active for 60 min according to the questionnaire.

### 3.3. Program Attendance

**Figure 1:**
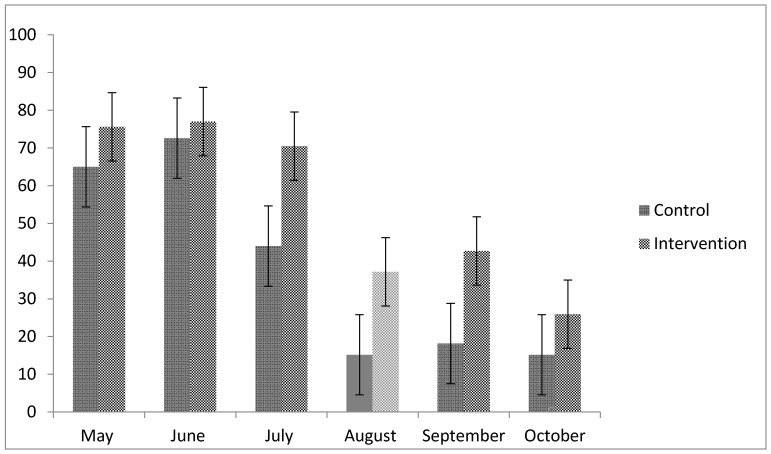
Percentage of high-intensity interval training (HIIT) exercise sessions attended (depicted with 95% confidence intervals). Note that summer vacation started at the beginning of August (lighter color to indicate summer vacation).

**Figure 2:**
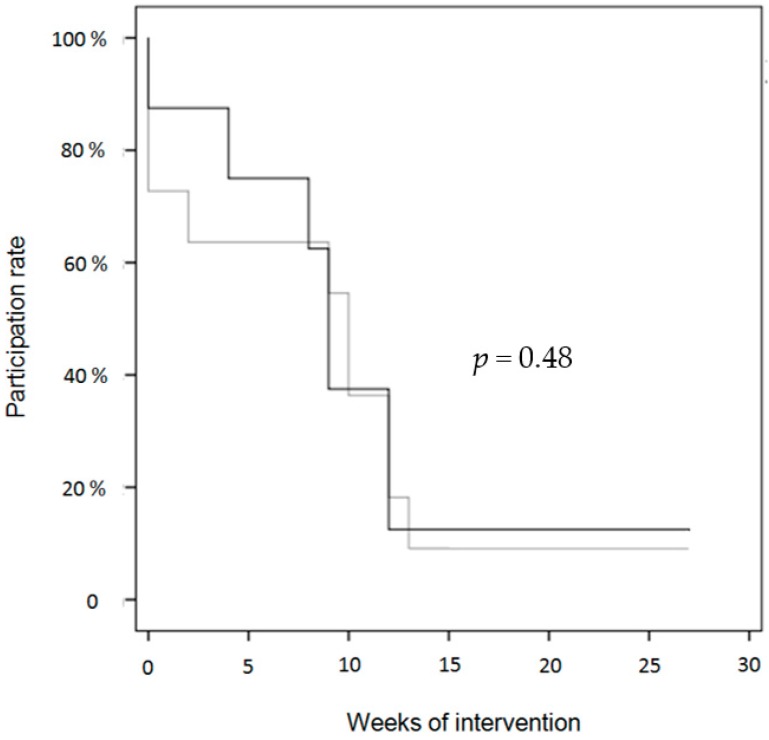
Kaplan-Meier survival analysis of drop-out over the time course of the intervention beginning in May at zero weeks. Grey line = control group; black line = intervention group.

Over a period of six months, the participants were supposed to attend two of three available exercise sessions per week for a total of 40 sessions. In the control group, 13.6 ± 10.7 (34%) sessions were attended on average compared to 19.5 ± 12.1 (49%) in the intervention group for a difference of 5.9 CI (−3.1, 15.0) sessions, *p* = 0.19. A sensitivity analysis with the Wilcoxon-Mann-Whitney U test yields the value *p* = 0.165. Program participation for the intervention and the control group is shown in percent for each month of the intervention (Figure 1). Participation within the first two months of the program ranged between 65% and 75%, but declined within the last 3 months of the training to a value as low as 15%. This drop coincided with the beginning of the school summer vacation and remained low after the start of the new school year. Survival analysis (log rank) showed that the drop-out between the groups was not significantly different (*p* = 0.48; χ^2^ = 0.49; see Figure 2) although the data show that the intervention group adhered to the program somewhat better in the first two months.

Reasons for dropout were categorized as school and leisure obligations (homework, after-school programs, etc.), mentioned by 65% of participants who terminated the program before the end of the intervention, followed by general appointments (doctor, administrative issues, etc.). A lack of interest in the program was provided by 20% of all participants as a reason for not attending subsequent training sessions.

### 3.4. Program Acceptance/Evaluation

Participants were asked to evaluate the program at the follow-up visit at the study center and the overall rating from the 20 participants who attended the follow-up visit (7 of whom completed the HIIT-training program) was 2.33 ± 1.37 on a scale from 1 (liked the program very much) to 10 (did not like the program at all). If the missing participant had all answered the question 2 standard deviation above the mean (i.e., chosen the answer 5) then the mean rating would still be 3.1.

The exercises themselves received the score 2.75 ± 1.55 on the same scale. The use of the website and the reception of text messages were evaluated as good by 60% of the participants, while 10% mentioned that they found the text-messages were sent too often to the participants. 

There was no correlation between program attendance and overall positive program evaluation by the participants themselves (*r* = 0.02, 95% CI (−0.59; 0.58), *p* = 0.942).

### 3.5. Anthropometric Parameters

Detailed information regarding the outcome of anthropometric parameters and cardiometabolic risk factors are provided in another analysis of the MoveHIT program [16]. Briefly, mean BMI-SDS was 2.33 ± 0.80 at baseline and decreased by 0.026 (95% CI −0.048 to 0.10) units after the intervention (*p* = 0.49). Waist circumference as marker of abdominal obesity decreased from 101 ± 13 cm by 1.8 (95% CI −0.07 to 3.6) cm, *p* = 0.071, and the related waist-to height ratio (WHtR) decreased from 0.596 ± 0.076 by 0.013 (95% CI 0.0025 to 0.024), *p* = 0.023. Body fat (BF) content according to Slaughter, which is based on skinfold measurements, decreased from 51.4% ± 7.5% by 2.0 (95% CI 0.58 to 3.5) percentage points, *p* = 0.011. The correlation between changes in WHtR and BF was 0.19 (−0.29, 0.59), *p* = 0.45, and was 0.21 (−0.26, 0.59), *p* = 0.39 and 0.51 (0.07, 0.78), *p* = 0.026 between changes in BMI-SDS and WHtR and BF-Slaughter, respectively.

## 4. Discussion

The purpose of this randomized controlled study was to test the feasibility of a six-month, media-supported HIIT program for overweight adolescents. We hypothesized that HIIT training is beneficial and efficient for overweight adolescents when motivation for regular attendance can be offered. This is one of the first studies to examine a media supported HIIT program regarding attendance and acceptance as well as anthropometric characteristics in overweight adolescents.

So far, continuous moderate exercise has been the main recommendation for physical exercise for losing or stabilizing weight [37,38]. HIIT has been shown to be especially time-efficient and to have positive results on the cardio-respiratory fitness and weight status in studies on adults [39,40,41,42]. Only limited data is available on the structure, implementation, and efficacy of a HIIT program directed at overweight adolescents. Attendance has been previously reported to be a crucial factor for perseverance of training participation, especially in HIIT programs [43].

In our feasibility study we experienced considerable difficulties in recruitment of participants and the planned number of overweight adolescents needed to participate in the study to fulfill power requirements could not be met. Given the data we observed and our true sample size of 14 per group, one can estimate a retrospective power of 26% for obtaining a confidence interval of the intended width. Even though we recruited through newspaper advertisements, schools, youth clubs, medical offices, and advertisement on public transport, response rates remained too low. Recruitment through a former obesity outpatient program was most successful as adolescents might have already been sensitized to that topic. Future studies need to establish more successful recruitment strategies for this target group. The American research group of Tate et al. found (mass) emailing to be the best cost-effective way of recruitment in obesity prevention, however, this needs to be examined in other cultural contexts [44]. By all means, multiple recruitment methods including electronic media should be considered to improve program registration in obesity prevention [45,46].

We showed that a six-month randomized controlled feasibility study had a relatively high program attendance during the first two months of participation. High initial attendance rates suggest that adolescents enjoyed the program at the start. However, there was also a rapid decline in participation during the following three months (as low as 15%). Furthermore, we observed that despite the high decline in participation, the program was overall rated as “good” at the end of the intervention. “School commitments” were main reasons for drop-out, even though drop-out occurred in the summer vacation. This could have been due to socially accepted answering behavior of the adolescents or due to new school obligations being imminent again, as the questionnaire on enjoyment of the program was filled out in October. A higher attendance in HIIT has been observed when incorporated into regular school curricula [12,47]. Mean attendance in a 10-week school HIIT program in the UK was 77% + 13% [48]. A decline divided into two phases of participation has been observed in the Loozit^®^ intervention, where initial attendance rates ranged around 85% and dropped to 47% [49].

In our case, attrition of attendance rates coincided with school holidays in Germany. Furthermore, school obligations, general appointments, and reluctance to participate were the most common issues when asked about reasons for drop-out. Previous studies have also documented that summer vacation can be a reason for discontinuing an intervention program, while lower educational status, a higher level of obesity, and residency in a tertiary service area [50,51], as well as logistical problems regarding time and location of training sessions [52] or a higher BMI-SDS [53], are reasons for program drop-out. Reports on program attendance rates are crucial information for the success and sustainability of future health promotion programs and need further attention in future large scale research [54].

The intervention through motivating text messages might have led to a slower drop-out of the program by the intervention group but the difference was not significant, which was also observed by Nguyen et al. where participants lost interest in these messages over time [49]. However, differences in attendance could also be attributed to other factors. Motivating messages could have initially encouraged adolescents through emphasizing self-efficacy and goal-setting, which has also been observed in adolescents and young adults attending a diabetes prevention program [55]. A web-based intervention with tailored physical activity counseling also showed a short-term effect on cardiorespiratory fitness, health-related quality of life, and BMI among overweight adolescents [56]. Furthermore, it has been shown that text messages can generally remind recipients to implement intentions and thereby increase exercise frequency [57]. Additionally, web-based social networking interventions delivered via social platforms can also increase short time physical activity change [58] Previous results have also shown that in addition to improved cardiorespiratory fitness, enjoyment of exercise and quality of life also increased significantly due to HIIT training [59]. Some of our study participants were more aware of the benefits of physical activity and the need to meet weekly physical activity recommendations better after the completion of the moveHIT program. However, sustainability and maintenance of this behavior need to be examined in future studies. 

Regarding anthropometric study outcomes, an improvement in some measures of body composition were observed, though the lack of correlation between some of them makes their interpretation difficult. Analyses of anthropometric measures, adipokines, and cardiometabolic risk factors of the MoveHIT study have been presented separately. The trial was not powered to demonstrate such changes, however, so a lack of significant results was to be expected.

Further limitations of this study are as follows. The two treatment groups of the study took part in the HIIT sessions together, meaning they could influence each other. The daily routine changed for participants as summer vacation was included in the time course of the intervention. To observe long-term changes in outdoor physical activity programs, there was no possibility to avoid this. A larger study setting would be necessary to allow for a design where this is not the case. The questionnaires were only filled out by 86% of the participants, meaning that respective results may be somewhat biased. A more precise evaluation of the effects of HIIT on the anthropometric measures and physiological effects would have been possible through a bigger sample size. The main strength of this study is that it is—to the best of our knowledge—the first study that has investigated high intensity interval training and its acceptance in overweight adolescents in a controlled setting. Training was offered for a period of six months including a possibility to prolong training, and a comprehensive set of data (participation rates, anthropometric and metabolic parameters, data on physiological performance, sedentary habits) were obtained.

## 5. Conclusions

Our study suggests that, ironically, although the HIIT program is “well-liked” by most participants, dropout rates are high. Media support may well improve attendance, though this could not be shown with certainty given that the recruitment difficulties resulted in an underpowered study. More effective recruitment strategies need to be implemented, since it proved to be difficult to attract participants. It is also important to conceive of ways to improve attendance throughout and beyond the summer vacation. Certain anthropometric parameters improved after the moveHIT program, however larger scale trials addressing these issues are needed to shed further light on the effectivity of HIIT.

## Figures and Tables

**Table 1:** Text message content over the course of the intervention.

Time	Month 1	Month 2	Month 3	Month 4	Month 5	Month 6
Media support (social cognitive theory) example message (shortened)	General attitude of physical activity *“Physical activity is beneficial for your health and well-being. The moveHIT program can help you to improve your fitness!”*	Outcome expectations *“By attending the moveHIT program twice a week, you can increase your muscle strength and feel more flexible.”*	Behavioral strategies to practice HIIT *“Write down the dates for the training in your calendar, to remind yourself to come to the moveHIT program.”*	Seeking social support for physical activity *“Tell your friends and family of the moveHIT program. They can join for a trial session and exercise with you.”*	Increasing self-efficacy in HIIT *“We have noticed your improvements in the training and you will have noticed that you can perform the exercises more easily now!”*	Practicing social cognitive constructs *“Think of the last months and keep reminding yourself of the training sessions and include your friends and family in your physical activity regimen.”*

## Abbreviations

The following abbreviations are used in this manuscript:

BMI	Body Mass Index
HIIT	High-intensity interval raining
WHtR	Waist-to-height ratio
